# Soluble TNF mediates amyloid-independent, diet-induced alterations to immune and neuronal functions in an Alzheimer’s disease mouse model

**DOI:** 10.3389/fncel.2023.895017

**Published:** 2023-03-15

**Authors:** Kathryn P. MacPherson, Lori N. Eidson, Madelyn C. Houser, Blaine E. Weiss, Jenna L. Gollihue, Mary K. Herrick, Maria Elizabeth de Sousa Rodrigues, Lindsey Sniffen, Erica M. Weekman, Adam M. Hamilton, Sean D. Kelly, Danielle L. Oliver, Yuan Yang, Jianjun Chang, Timothy R. Sampson, Christopher M. Norris, Malú Gámez Tansey

**Affiliations:** ^1^Department of Physiology, Emory University School of Medicine, Atlanta, GA, United States; ^2^Nell Hodgson Woodruff School of Nursing, Emory University, Atlanta, GA, United States; ^3^Sanders-Brown Center on Aging, University of Kentucky College of Medicine, Lexington, KY, United States; ^4^Department of Neuroscience and Center for Translational Research in Neurodegenerative Disease, The University of Florida College of Medicine, Gainesville, FL, United States; ^5^Department of Cell Biology, Emory University School of Medicine, Atlanta, GA, United States

**Keywords:** soluble TNF, Alzheimer’s disease, neuroinflammation, T cells, macrophage, electrophysiology, flow cytometry, microbiome

## Abstract

**Introduction:** Increasing evidence indicates that neurodegenerative diseases, including Alzheimer’s disease (AD), are a product of gene-by-environment interplay. The immune system is a major contributor mediating these interactions. Signaling between peripheral immune cells and those within the microvasculature and meninges of the central nervous system (CNS), at the blood-brain barrier, and in the gut likely plays an important role in AD. The cytokine tumor necrosis factor (TNF) is elevated in AD patients, regulates brain and gut barrier permeability, and is produced by central and peripheral immune cells. Our group previously reported that soluble TNF (sTNF) modulates cytokine and chemokine cascades that regulate peripheral immune cell traffic to the brain in young 5xFAD female mice, and in separate studies that a diet high in fat and sugar (HFHS) dysregulates signaling pathways that trigger sTNF-dependent immune and metabolic responses that can result in metabolic syndrome, which is a risk factor for AD. We hypothesized that sTNF is a key mediator of peripheral immune cell contributions to gene-by-environment interactions to AD-like pathology, metabolic dysfunction, and diet-induced gut dysbiosis.

**Methods:** Female 5xFAD mice were subjected to HFHS diet for 2 months and then given XPro1595 to inhibit sTNF for the last month or saline vehicle. We quantified immune cell profiles by multi-color flow cytometry on cells isolated from brain and blood; metabolic, immune, and inflammatory mRNA and protein marker biochemical and immunhistological analyses, gut microbiome, and electrophysiology in brain slices were also performed.

**Results:** Here, we show that selective inhibition of sTNF signaling via the biologic XPro1595 modulates the effects of an HFHS diet in 5xFAD mice on peripheral and central immune profiles including CNS-associated CD8+ T cells, the composition of gut microbiota, and long-term potentiation deficits.

**Discussion:** Obesogenic diet induces immune and neuronal dysfunction in 5xFAD mice and sTNF inhibition mitigates its effects. A clinical trial in subjects at risk for AD due to genetic predisposition and underlying inflammation associated with peripheral inflammatory co-morbidities will be needed to investigate the extent to which these findings translate to the clinic.

## 1 Introduction

Neurodegenerative diseases such as Alzheimer’s disease (AD) are a product of gene-by-environment interactions within the context of an individual’s genetics, lifestyle choices, and environmental exposures. A major arbiter of these effects is the immune system, and there is mounting evidence that peripheral inflammation is linked to an increased risk of developing AD. Increased levels of cytokines, including tumor necrosis factor (TNF), accelerate cognitive decline and transition from mild cognitive impairment to AD (Holmes et al., 2003, 2009). Conditions associated with low-grade chronic peripheral inflammation such as metabolic syndrome, insulin resistance, diabetes, and obesity have all been linked to increased risk for AD (Dahlgren and Magnusson, 1980; Ojo and Brooke, 2015; Walker and Harrison, 2015; De Sousa Rodrigues et al., 2019). Recently, emphasis has been placed on identifying early life risk factors and biomarkers likely to contribute to the development of AD to identify at-risk individuals and opportunities for disease prevention. It is estimated that over 34% of Americans over age 20 have metabolic syndrome, defined as abdominal obesity with a combination of high triglycerides, lipoprotein, blood pressure, or fasting glucose levels (Yates et al., 2012; Saklayen, 2018). Conditions associated with the consumption of an unhealthy diet may therefore represent an opportunity for early intervention through the modulation of inflammatory mechanisms that mediate the gene-by-environment effect in AD. Consumption of a high-fat diet or a high-carbohydrate diet induces chronic low-grade systemic inflammation, deficits in cognition, and disruptions of gut bacteria leading to increased proinflammatory cytokine production and degeneration of gut epithelium integrity (Proctor et al., 2017; Satokari, 2020; de Paula et al., 2021). The presence or absence of specific gut microbiota affects microglial maturation and function, supporting the role of a healthy gut in a healthy brain environment (Erny et al., 2015). In addition, microbial metabolites can reprogram monocytes into macrophages with either pro-inflammatory or anti-inflammatory activity that traffic to the central nervous system (CNS) and its borders in health and in AD (van de Wouw et al., 2019). Human studies of adolescents and adults have linked metabolic syndrome and insulin resistance to decreased executive function and declarative memory, while animal studies have linked obesity and high-fat diet to increased amyloid β (Aβ) levels, indicating a mediating role for diet-induced peripheral inflammation in AD (Yates et al., 2012; Radler et al., 2015; Wakabayashi et al., 2019). Peripheral inflammation, including that associated with a high-fat diet, increases blood-brain barrier (BBB) leakiness and microglia activation (Sheikh et al., 2022). Changes in the regulation of peripheral immune cell traffic due to a leaky BBB may be a contributor to diet-induced neuroinflammation and acceleration of AD-like pathology. It remains unclear how low-grade chronic inflammation conditions contribute to increased AD risk, although they may work through an alteration in trafficking patterns of peripheral immune cells to the brain. Evidence from both patients and animal models suggests that at terminal stages of neuroinflammatory diseases such as AD, populations of peripheral immune cells are present within the parenchyma (Togo et al., 2002; Rezai-Zadeh et al., 2009; Cao and Zheng, 2018).

Our group reported that trafficking patterns of different populations of peripheral immune cells to the CNS of 5xFAD mice have varying time courses that could indicate adaptive as well as maladaptive responses that contribute to the chronic neuroinflammation associated with amyloid plaque deposition. More importantly, we also demonstrated that sTNF signaling plays a role in modulating these trafficking patterns (MacPherson et al., 2017). In addition, our group independently demonstrated that a high-fat, high-sugar (HFHS) diet disrupts inflammatory gene networks in the brain, liver, and gut of wild-type C57BL/6J mice and promotes behavioral deficits in rodents, and that sTNF is a key cytokine driving these processes (de Sousa Rodrigues et al., 2017), raising the interesting and therapeutically relevant possibility that sTNF plays a central role in linking mechanisms of diet-induced peripheral inflammation that contribute to neuroinflammation in AD and disease progression.

Therefore, the primary purpose of this study is to establish the extent to which an HFHS diet further disrupts central and peripheral immune cell profiles, peripheral (blood) inflammation, gut microbiome, and synaptic dysfunction in the 5xFAD mouse model of AD-like pathology and the impact of selective sTNF inhibition with XPro1595 on these phenotypes.

## 2 Material and methods

### 2.1 Animals

The 5xFAD (Tg) mouse model develops pathological hallmarks of AD, with amyloid pathology beginning at 2.5–3 months of age (Oakley et al., 2006). Female 5xFAD mice have accelerated AD-like pathology compared with males (Oakley et al., 2006), and thus 2–2.5 month-old adult female 5xFAD mice and non-Tg littermate control mice were bred in a facility at Emory University and utilized for this study. Mice were co-housed (Tg and non-Tg together, CD and HFHS diet-fed mice in different cages, XPro1595 and Saline groups not separated by cages) in groups of 3–5 with *ad libitum* access to food and water on a 12:12 light/dark cycle (lights on at 7A.M.). Mice for flow cytometry, immunohistology, and biochemistry were housed in a vivarium at Emory University; mice for electrophysiology were housed in a vivarium at the University of Kentucky in standard cages under the 12-h light/dark cycle with *ad libitum* access to food and water (see Section 2.3 *Diet*). At 3–3.5 months of age, mice were given a subcutaneous injection of the novel sTNF biologic XPro1595 or vehicle (0.8% saline) for one month until the end of the diet period. The initial size of cohorts was large (*n* = 20) to power the more variable outcome measures based on previously published data, but some attrition was experienced over time. The majority of the analyses required an n of 7 for 80% power and alpha set at 0.05. All procedures were approved by the Institutional Animal Care and Use Committee of Emory University or University of Kentucky and complied with the National Institute of Health *Guide for the Care and Use of Laboratory Animals*.

### 2.2 Genotyping

The genotypes of 5xFAD mice were determined by PCR analysis of tail DNA to detect human APP and PS1 genes. The following primers from Jackson lab were used: APP Forward 5’-AGGACTGACCACTCGACCAG-3’, APP Reverse 5’-CGGGGGTCTAGTTCTGCAT-3’; PS1 Forward 5’- AATAGAGAACGGCAGGAGCA-3’, PS1 Reverse 5’-GCCATGAGGGCACTAATCAT-3’. The amplification conditions were 94°C for 3 min followed by 35 cycles with denaturation at 94°C for 30 s, annealing at 52°C for APP (60°C for PS1) for 1 min, and elongation at 72°C for 1 min. The PCR products were separated on a 2% agarose gel by electrophoresis.

### 2.3 Diet

At 2–2.5 months of age, 5xFAD and non-Tg littermate female mice were fed for 2 months (8 weeks) with either a control diet (CD; TD.150112, 15% kcal fat, ENVIGO, Indianapolis IN) or a high-fat high-sugar diet (HFHS; TD.150111, 40% kcal fat, 45% kcal carbohydrate, ENVIGO, Indianapolis IN; Table 1). Because mice from each group were group-housed to avoid cohort effects, food intake was not assessed. Food was replenished twice a week. Mice were weighed weekly.

**Table 1:** Control (CD) and high-fat high-sugar (HFHS) diet composition.

**Ingredient (g/Kg)**	**TD.150111**	**TD.150112**
Casein	180.00	180.00
L-Cystine	3.00	3.00
Corn Starch	31.00	404.49
Maltodextrin	95.46	155.00
Sucrose	100.00	100.00
Fructose	290.00	0.00
Anhydrous Milkfat	185.00	10.00
Beef Tallow	18.00	0.00
Cellulose	50.00	50.00
Soybean oil	0.00	50.00
Mineral Mix, AIN-93G-MX (94046)	35.00	35.00
Vitamin Mix, Teklad (40060)	10.00	10.00
Choline Bitartrate	2.50	2.50
TBHQ, antioxidant	0.04	0.008

### 2.4 XPro1595 administration

**Figure 1:**
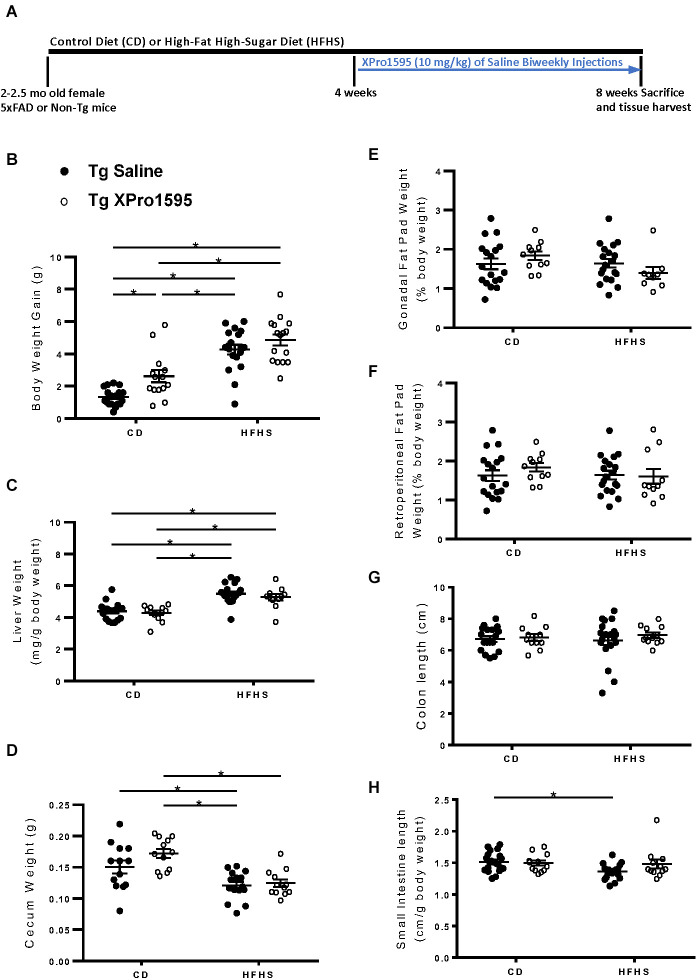
Experimental timeline and effects of HFHS diet and sTNF inhibition on body, liver, and fat pad weight in 5xFAD female mice. **(A)** Experimental timeline. **(B)** Body weight gain at end of the study from baseline (*n* = 11–22 mice per group). **(C)** Liver weight over body weight (mg/g; *n* = 10–20 mice per group). **(D)** Cecum weight in grams (*n* = 8–18 mice per group). **(E)** Gonadal fat pad weight as a percentage of body weight (*n* = 10–20 mice per group). **(F)** Retroperitoneal fat pad weight as a percentage of body weight (*n* = 9–20 mice per group). **(G)** Colon length (cm; *n* = 10–20 mice per group). **(H)** Small intestine length over body weight (cm/g; *n* = 10–21 mice per group). Asterisks indicate significant (*p* < 0.05) differences by Tukey’s multiple comparisons test between groups following two-way ANOVA. Saline vehicle (black circles), XPro1595 (white circles). ns = not significant.

XPro1595 is a PEG-ylated human TNF variant devoid of TNF receptor-binding activity that due to its mechanism of action only forms heterotrimers with native soluble TNF (sTNF) but not membrane-bound TNF (tmTNF), thereby sequestering sTNF away from TNF receptors (Steed et al., 2003). When administered peripherally, XPro1595 is detectable in the brain at levels sufficient to neutralize native soluble TNF. XPro1595 (generously provided by Dr. David Szymkowski at Xencor, Inc.) was diluted with sterile saline to 2 mg/ml before dosing. Starting 4 weeks after the start of the diet, mice received either the brain-permeant biologic XPro1595^®^ (10 mg/kg) or vehicle (1 ml/kg) by subcutaneous (sc) injection twice a week (every third day) for 4 weeks for a total of eight injections until the end of the diet intervention (Figure 1A). The XPro1595 dose was selected based on PK/PD studies and previously published work (MacPherson et al., 2017).

### 2.5 Experimental timeline, euthanasia, and tissue collection

At 4–5 months of age and 8 weeks after the start of the diet regimen, mice for tissue collection were cervically dislocated under isoflurane anesthesia. Gonadal and retroperitoneal fat pads and liver were removed and weighed immediately. Large and small intestines were dissected, measured, and cleaned. A 1 mm section of the distal duodenum, jejunum, ileum, and colon were flash-frozen in liquid nitrogen and stored at −80°C. Brains were hemisected on ice, and the right side was immediately processed for flow cytometry analysis or the hippocampus was microdissected on a petri dish cooled with dry ice, flash-frozen in 2-methyl-butane on dry ice, and stored at −80°C until processing for qPCR. Left brain hemispheres were post-fixed in 4% paraformaldehyde (PFA). Liver, retroperitoneal, and gonadal adipose tissue weight and small intestine length were normalized by body weight. Blood (*via* cheek bleeds prior to euthanasia) and feces were collected. Feces and plasma (isolated from whole blood) were flash-frozen in liquid nitrogen and stored at −80°C until assayed. PBMCs in whole blood were immediately stained for flow cytometry analysis.

### 2.6 Quantitative PCR

RNA was extracted using the RNeasy Mini Kit (Qiagen, Hilden Germany) according to the manufacturer’s instructions. RNA was purified with DNAse I (Life Technologies; 0.64 μl per 4 μg) for 30 min at 37°C then 10 min at 75°C and its concentration was measured using a Nano-Drop 2000 spectrophotometer and standardized to 4 μg. RNA was converted to cDNA using Super-Script II Reverse Transcriptase (Life Technologies), quantified *via* PicoGreen assay (Life Technologies, Carlsbad, CA), and standardized to 1 μg. Samples were run in triplicate [coefficient of variation (CV) 4%]. Fold changes in brain gene expression over non-Tg+CD+saline were calculated by the comparative 2^−ΔΔCT^ quantification and normalized to *Gapdh* or *Tbp*.

The following primer sequences were designed and purchased from Integrated DNA Technologies (IDT). *Cldn2* (Forward- CAC CCA CAG ATA CTT GTA AGG, Reverse- AGC CTC TAA TCC CTT ATT TCA C), *Zo-1* (Forward- CCT GAA GGA ATT GAG CAA GA, Reverse- GCA GAG TTT CAC CTT TCT CT), *Ccl2* (Forward- CAC CCA CAG ATA CTT GTA AGG, Reverse- AGC CTC TAA TCC CTT ATT TCA C), *Tnf* (Forward- CTG AGG TCA ATC TGC CCA AGT AC, Reverse- CT CAC AGA GCA ATG ACT CCA AAG), *Cd45* (Forward- TCA TGG TCA CAC GAT GTG AAG A, Reverse- AGC CCG AGT GCC TTC CT), *Il-6* (Forward- GAG GAT ACC ACT CCC AAC AGA CC, Reverse- AAG TGC ATC ATC GTT GTT CAT ACA), *Gapdh* (Forward- GAG GTG ACC GCA TCT TCT TG, Reverse- CCG ACC TTC ACC ATC TTG TC), *Tata* (Forward-GTA TCT GCT GGC GGT TTG G, Reverse-GGC ACT GCG GAG AAA ATG A).

### 2.7 Metabolic and immune assays

Whole blood (200 μl) was collected into vacutainers containing EDTA. Fifty microliters of blood were transferred into a 15 ml conical tube on ice for immune cell phenotyping *via* flow cytometry. The remainder of the blood was centrifuged at 3,000× *g* for 12 min at 4°C; plasma was stored at −80°C. Cholesterol was measured using the spectrophotometric Cholesterol Quantitation Kit (#MAK043, Sigma-Aldrich, St. Louis, MO). Plasma insulin and leptin were measured using the Mouse Metabolic Kit (Multi-spot Assay System # K15124C-1, Meso Scale Discovery, MSD, Rockville, MD). LCN2 was assessed using the Mouse Lipocalin-2/ NGAL Quantikine ELISA Kit, #MLCN20, R&D Systems Minneapolis, MN). The Mouse Proinflammatory 7-Plex Ultra-Sensitive Assay (Kit #K15012C-1, MSD, Rockville, MD) was used to assess inflammatory cytokines. For multiplexed immunoassays, plasma was diluted 1:4 using the MSD 1X homogenization buffer, and assays were performed per manufacturer’s instructions on the MSD SECTOR Imager 2400-A (Meso Scale Diagnostics, LLC, Rockville, MD). Intestinal tissue was homogenized with cold stainless-steel beads on the Qiagen TissueLyser II for 2–5 min at 20 Hz in phosphate buffered saline (PBS) containing 0.1% Tween 20 to a final concentration of 100 mg/ml. Samples were then centrifuged for 10 min at 3,000× *g* at 4°C. Supernatants were collected, and total protein was assayed using a Pierce BCA protein kit and Spectramax plate reader at 562 nm using SoftMax v5.2 software (Molecular Devices, CA). LCN2 was immediately estimated in the supernatants. Plasma and intestinal tissue samples were assayed in duplicate by a blinded experimenter.

### 2.8 Brain dissociation for immune cell isolation

Half brain (hemisected through the sagittal plane) tissue was finely minced in 1× Hanks’ Balanced Saline Solution (HBSS; without calcium, magnesium, and phenol red, Invitrogen 14175) and transferred to an enzymatic Dipeptidyl Peptidase (DDP) solution [Dulbecco’s Modified Eagle Medium (DMEM)/F12 media containing 1 mg/ml papain from papaya latex (P4762 Sigma Aldrich, St. Louis, MO), 1.2 U/ml dispase II (4942078001 Roche diagnostics, Risch-Rotkreuz, Switzerland), and 220 U/ml DNAse I (18047-019 Invitrogen, Carlsbad, CA)]. Minced tissue in DDP was incubated at 37°C for 20 min before the enzymatic activity was neutralized with 10% Fetal Bovine Serum (FBS; heat-inactivated, Atlanta Biologicals, S11150). Brain tissue was centrifuged for 5 min at 2,000× *g*, and the pellet was homogenized further in ice-cold 1× HBSS using a polished, fine-tip glass pipette. The homogenate was filtered through a 70-μm cell strainer. The pellet was resuspended in 37% Percoll (Percoll pH 8.5–9.5; Sigma Aldrich Co, P1644), and 70% Percoll was layered below, 30% Percoll was layered above, and 1× HBSS was layered on top. The Percoll gradient was centrifuged at 500× *g* without brake, and immune cells were collected from the buffy coat between the 70% and 37% Percoll layer and washed with 4× volume of 1× HBSS. Cells (approximately 1 × 10^6^) were stained with an antibody panel in preparation for flow cytometry.

### 2.9 PBMC collection

To isolate peripheral blood mononuclear cells (PBMCs), 50 μl of blood was incubated with 1 ml red blood cell lysis buffer (BioLegend cat# 420301) at room temperature in the dark for 10 min and then spun at 3,000× g for 5 min at 4°C. The pellet was washed with 5 ml 1× HBSS and spun again. The pellet was resuspended in 200 μl of 1× PBS and stained for flow cytometry.

### 2.10 Multi-color flow cytometry

Brain immune cells and PBMCs were stained with Live/Dead Fixable Aqua (1:2,000, L34957 Invitrogen) and incubated with anti-mouse CD16/CD32 (Fc block; 1:100 140161085 eBioscience). Cells were stained with the following antibody panel: anti-mouse Ly6G Pacific Blue (1:100, Biolegend 127612), CD11b Pe-Cy7 (1:200, Biolegend 101215), CD4 PE (1:100, eBiosciences 12-0041-81), CD8b APC_e780 (1:100, eBiosciences 47-0083-82), CD45 PerCP-Cy5.5 (1:100, eBiosciences 45-0451-82), CD3 PE 610 (1:100, eBiosciences 61-0031-82), CD11c AlexaFluor 700 (1:50, Biolegend 117320), MHCII APC (1:50, Miltenyi 130-102-139), and Ly6C FITC (1:200, eBiosciences 53-5932-82) in FACS buffer. Samples were run on an LSR II (BD Biosciences) and analyzed with FlowJoV10. Single cell lymphocytes were gated based on forward scatter height (FSH; size) and side scatter height (SSH; granularity) and then by FSH by forward scatter area (FSA). Live cells were selected as the Fixable Aqua negative population. CD45^+^ cells were selected and then gated for CD11b and CD3. CD11b^+^CD3^−^ (myeloid cells) were gated for CD11c and Ly6G. CD11c^−^Ly6G^−^ (monocytes/microglia) were gated for CD45 low and high, and each population was further gated for Ly6C and MHCII low or high based on histogram distribution. CD11c^+^Ly6G^−^ cells were considered dendritic cells and CD11c^−^Ly6G^+^ cells were considered neutrophils or potentially a subpopulation of antigen-presenting microglia (Wlodarczyk et al., 2014). As described in a previous publication from our group (MacPherson et al., 2017), CD11b^+^ CD45^high^ cells were classified as peripheral macrophages which had trafficked into the brain while CD11b^+^ CD45^low^ cells were classified as microglia. CD11b^−^CD3^+^ cells (T cells) were gated on CD4 and CD8b to distinguish helper T-cell populations and cytotoxic T cell populations, respectively. Counting beads (AccuCheck Counting Beads, Invitrogen PCB100, 10 μl per sample) were selected on the size from FSH vs. FSA plot. Sample counts of all target populations were calculated in FlowJo, and total tissue counts were obtained using the formula [(population count/bead count) × (bead concentration in sample run) × (run volume) × (whole sample volume/volume processed for 1 × 10^6^ cells)]. Compensation controls were run with each cohort.

### 2.11 Immunohistochemistry

For all mice, brain hemispheres were flash-frozen and sectioned at 25 μm on a freezing microtome and stored in cryoprotectant. Whole brain was labeled separately with primary antibodies against IBA-1 (Waco Catalog#: 019-19741; rabbit anti-mouse; 1:1,000), GFAP (Thermofisher Catalog#: 13-0300; rat anti-mouse; 1:1,000), and amyloid-β (Chemicon Catalog# MAB1560 Ab 6E10 @ 1:500). The sections were washed in 0.01 M PBS. Then they were agitated in a 3% hydrogen peroxide solution and subsequently washed in PBS and then tris-buffer saline (TBS). Sections were blocked for an hour with 8% normal goat serum for GFAP and Iba1 and 8% normal horse serum for amyloid-β, avidin, and Triton-X (1:100) in TBS for 1 h at 4°C. Sections were then incubated at 4°C with gentle agitation for approximately 24 h in the respective primary antibody, biotin, and 2% normal goat or horse serum in TBS. The following day, the sections were washed in TBS and then agitated for 1 h at 4°C in a secondary biotinylated antibody solution with 2% normal goat or horse serum in TBS. The Iba1 sary antibody used was goat anti-rabbit (Vector Catalog#: BA1000; 1:200), the GFAP secondary antibody used was goat anti-rat (Vector Catalog#: BA9400; 1:200), and the amyloid-β secondary antibody used was horse anti-mouse (Vector Catalog#: BA2001; 1:200). Sections were washed in TBS, incubated in Vectastain ABC Elite Kit for 1 h at 4°C, then washed in TBS again. Subsequently, sections were incubated for 3 min in Sigma 3,3’ Diaminobenzidine (DAB) tablet solution, and then washed and mounted.

The hippocampus and subiculum were analyzed for three regions using Nikon Eclipse 90i. For the hippocampus, three images were taken of each of the CA1, CA3, and dentate gyrus (DG) regions. For the subiculum, two images were taken each of the posterior, ventral, and dorsal regions. Images were taken at 2× , with an exposure of 1 ms, a gain of 1.00× , high contrast, and full constant light. Images were analyzed for optical density with ImageJ using the Rodbard function with a threshold set at 0.6 for all stains.

For amyloid confirmation analysis, 5–7 40 μm thick coronal cryosections spaced 480 μm apart spanning the hippocampus were cut on a freezing microtome and immunostained using an antibody against Aβ (rabbit polyclonal Aβ1–16, Life Technologies, Carlsbad, CA, USA). Immunohistochemistry was performed as previously described (Wilcock et al., 2008). Briefly, sections were quenched for endogenous peroxidase, blocked and permeabilized, then incubated overnight in the primary antibody (Aβ 1:1,000). After washing, sections were incubated for two hours in a biotinylated secondary antibody (goat anti-rabbit IgG, 1:3,000, Vector Laboratories, Burlingame, CA, USA). Sections were then washed and incubated for one hour in ABC (Vector Laboratories); then color development was performed using the ImmPACT DAB kit (Vector Laboratories). Sections were mounted, air-dried overnight, dehydrated, and coverslipped in DPX (Electron Microscopy Sciences, Hatfield, PA, USA). Slides were scanned using the Zeiss Axio Scan.Z1 Slide Scanner (Carl Zeiss Microscopy, Jena, Germany). Immunohistochemical analysis was performed using the Indica Labs HALO Object Colocalization v1.3 module (Albuquerque, NM, USA). For each section, separate ROIs were drawn around the cortex and hippocampus for analysis.

### 2.12 Electrophysiology measures in area CA1 of *in situ* brain slices

At 5 months of age, female 5xFAD mice were euthanized under isoflurane inhalation by decapitation at the University of Kentucky. Brain tissue was rapidly harvested using protocols described previously (Mathis et al., 2011). Brain slices of approximately 450 μm thickness were prepared on a Vibratome (Leica) in oxygenated (95% O_2_/5% CO_2_), ice-cold, Ca^2+^-free artificial cerebral spinal fluid (ACSF). Slices were then incubated for at least 3 h in warmed (30°C–32°C), oxygenated ACSF (CaCl_2_ added). Slices used for experiments were transferred to one of two Kerr Tissue Recording Systems [Kerr Scientific Instruments (KSI), Auckland, New Zealand] where they were submerged and perfused with oxygenated ACSF at a rate of approximately 2 ml/min (30°C–32°C). ACSF contained (in mM): 124 NaCl, 2 KCl, 1.25 KH_2_PO_4_, 2 MgSO_4_, 26 NaHCO_3_, 2 CaCl_2,_ and 10 dextrose, pH 7.4. Synaptic strength and plasticity measurements were recorded in *stratum radiatum* in area CA1 of the hippocampus according to our previous methods (Pleiss et al., 2016; Sompol et al., 2017). Stimulation (100 μs duration biphasic pulses) was delivered through a bipolar platinum iridium wire (placed in *stratum radiatum*, near the CA3-CA1 border) using a constant-current stimulus isolation unit (World Precision Instruments, Sarasota, FL). Field potentials were acquired from CA1 stratum radiatum using Ag-AgCl wire. Field potentials were filtered at 1 kHz. Stimulus timing and data acquisition were controlled by a PowerLab 4/35 data acquisition system (ADInstruments, Sydney, Australia) and Labchart software (ADInstruments). For synaptic strength and paired-pulse facilitation measures, double pulses, set at 50 ms apart, were delivered at a frequency of 0.1 Hz. Stimulus pairs were delivered at 12 intensities ranging from 25 to 500 μA, and 3 sets of field potentials were recorded and averaged per stimulus level to construct input/output curves. Afterwards, stimulation intensity was readjusted to elicit a field excitatory postsynaptic potential (fEPSP) between 0.5 and 1 mV. No treatment or diet differences were noted for baseline fEPSP slope amplitudes. Single pulse stimulation then commenced at a frequency of 0.033 Hz for at least 20 min to obtain a stable baseline. Long-term potentiation (LTP) was induced using two 1-s duration, 100 Hz stimulus trains delivered 10 s apart. Field potentials were then recorded for 1 h post-tetanic stimulation.

### 2.13 Microbiome analysis

Mice were co-housed (Tg and non-Tg together, CD and HFHS diet-fed mice in different cages, XPro1595 and Saline groups not separated by cages). Fecal pellets were collected immediately prior to sacrifice while the mouse was placed in a glass beaker, flash-frozen, and stored at −80°C until processing. Bacterial genomic DNA was extracted from the fecal pellets, quantified, and purified, and then 16S rRNA V3-V4 gene sequencing was performed by the Emory Genomics Core. Negative, no template control (NTC), positive, and Zymo mock microbial community controls were included in runs. Demultiplexed raw amplicon (16S) sequences in FastQ file format were processed, denoised, and dereplicated, including chimera removal and trimming of reads based on quality scores, using the “dada2” package (Callahan et al., 2016). One sample yielded poor quality data (13.1% of sequences passing quality thresholds) and was excluded from further analysis. Taxonomic composition analysis was performed by comparison against the Silva databases (v132). Sequences associated with contaminants (10) were identified and removed using the “decontam” package (Davis et al., 2017) based on their prevalence in the negative control samples. The “phyloseq” package (McMurdie and Holmes, 2013) was used to evaluate bacterial composition including Shannon and inverse Simpson measures of alpha diversity and Bray-Curtis dissimilarity for beta diversity (non-metric multidimensional scaling [NMDS] method). As no differences in diversity metrics were observed between 5xFAD and non-Tg mice, genotypes were combined for analysis of microbiome data. Lack of genotype effect was confirmed by regression analysis. After filtering taxa with counts of five or fewer in the whole data set, the “DESeq2” package (Love et al., 2014), which implements a negative binomial model, was used to identify taxa that differed between experimental groups (diet accounting for treatment and treatment accounting for diet, genotypes combined).

### 2.14 Statistical analysis

For data with four groups, means were compared using two-way ANOVAs and Tukey’s multiple comparisons test; *t*-tests were used to compare data sets with two groups, and one-way ANOVAs were used to compare three groups (GraphPad Prism). Diet and treatment effects on fEPSP/FV ratios and LTP amplitudes (averaged between 55 and 60 min post 100-Hz stimulation) were compared using two-way ANOVA and Fisher’s LSD test. One sample *t*-tests were used to determine if significant LTP was observed in each treatment group following delivery of 100 Hz stimulation. 16S sequencing data were analyzed using R (R Core Team, 2020) in the RStudio Team 2020 environment (RStudio Team RStudio: Integrated Development for R. RStudio, Inc., Boston, MA URL^1^) with additional packages “tidyverse” (Wickham et al., 2019) and “ggpubr” (Alboukadel, 2020). Alpha diversity comparisons by diet and by treatment (genotypes combined) were assessed by two-way ANOVAs and Tukey’s multiple comparisons test. Associations between alpha diversity and the experimental factors treatment, diet, and genotype were confirmed by linear regression (alpha diversity measure ~ Treatment + Diet + Genotype). Associations between beta diversity and treatment, diet, and genotype were evaluated by PERMANOVA (adonis2, “vegan” package (Oksanen et al., 2020) with 999 permutations.

## 3 Results

### 3.1 HFHS diet induces signs of metabolic syndrome and peripheral inflammation

**Figure 2:**
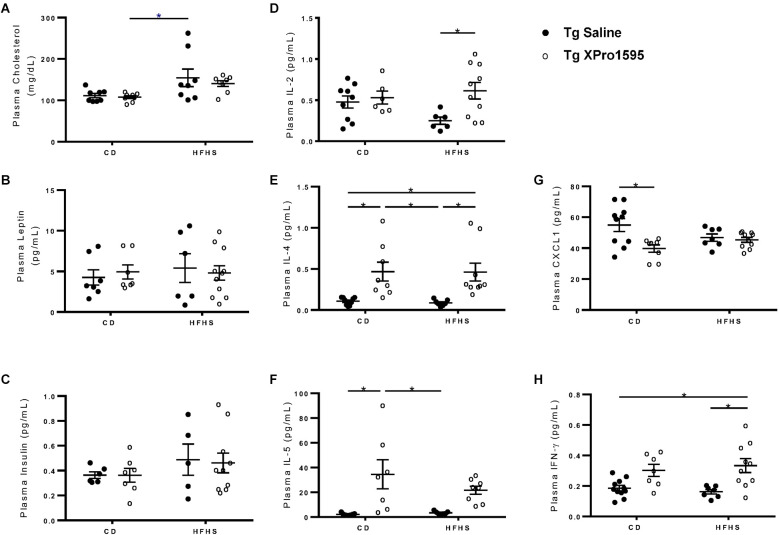
Plasma levels of analytes measured by immunoassays in Tg female 5xFAD mice.** (A)** Cholesterol, **(B)** Leptin, **(C)** Insulin, **(D)** IL-2, **(E)** IL-4, **(F)** IL-5, **(G)** CXCL1, **(H)** IFNγ levels in plasma from Tg mice on CD or HFHS diet treated with saline or XPro1595 (*n* = 7–10 per group). Asterisks indicate significant (*p* < 0.05) differences by Tukey’s multiple comparisons test between groups following two-way ANOVA.

First, 2–2.5 month old female 5xFAD or non-Tg mice were administered a Control Diet (CD) or High-Fat High-Sucrose (HFHS) diet, and we assessed general measures of fat and weight in the presence or absence of XPro1595, a soluble TNF-specific inhibitor delivered 4 weeks into the diet regimen (Figure 1A). We found that the HFHS diet increased body weight gain in 5xFAD mice compared to the control diet (CD; Figure 1B), as observed in previous studies (De Sousa Rodrigues et al., 2019). Inhibition of sTNF with XPro1595 increased weight gain in CD-fed mice (Figure 1B; main effect of diet *p* < 0.0001 and XPro1595 *p* = 0.002). The HFHS diet also increased liver weight, an indicator of ectopic lipid deposition (Longo et al., 2019), as also seen in previous studies (De Sousa Rodrigues et al., 2019), which was unaltered by sTNF inhibition (Figure 1C; main effect of diet *p* < 0.0001). Cecum weight was decreased by HFHS diet compared to CD in saline-treated mice (Figure 1D; main effect of diet *p* < 0.0001).Gonadal fat pad weight (Figure 1E), retroperitoneal fat pad weight (Figure 1F), and colon length (Figure 1G) were not significantly changed by the HFHS diet, but small intestine length was significantly decreased in saline-treated transgenic mice (Figure 1H; main effect of diet *p* = 0.05). Inhibition of sTNF decreased this elevated cholesterol in the HFHS diet-fed mice to levels statistically indistinguishable from the CD saline treated mice (Figure 2A), which was also observed in previous studies (De Sousa Rodrigues et al., 2019). No statistical differences in leptin (Figure 2B) and insulin (Figure 2C) were observed in the Tg mice as a function of diet or with inhibition of sTNF. Together, these changes show modulation of peripheral markers of metabolic syndrome induced by the HFHS diet. No significant differences were found in mRNA expression of CCL2, TNF, TGFβ, CLDN2, OCLN, or TJP-1, markers associated with inflammation and loss of barrier integrity in the gut (data not shown).

To further understand the extent of inflammation induced by the HFHS diet and reversed by sTNF inhibition, we also assessed plasma cytokine levels. Inhibition of sTNF significantly increased IL-2 in Tg HFHS mice (Figure 2D, main effect of XPro1595 *p* = 0.024), increased IL-4 independent of diet treatment (Figure 2E, main effect of XPro1595 *p* = 0.0001), and increased IL-5 (Figure 2F, main effect of XPro1595 *p* = 0.0001) in mice on the CD with a similar trend in mice on the HFHS diet. Inhibition of sTNF decreased CXCL1 in Tg mice on the CD but not on the HFHS diet (Figure 2G, main effect of XPro1595 *p* = 0.085 and interaction *p* = 0.02), and increased IFNγ in Tg mice on the HFHS (Figure 2H, main effect of XPro1595 *p* = 0.02). These data show that the use of the HFHS diet in our hands in female 5xFAD Tg mice is similar to the effect in non-Tg mice previously reported by our group (De Sousa Rodrigues et al., 2019) and that peripheral administration of the sTNF-specific inhibitor XPro1595 has systemic immunomodulatory effects in Tg mice.

### 3.2 HFHS diet and inhibition of sTNF influence peripheral blood immune cell profiles of 5xFAD mice

**Figure 3:**
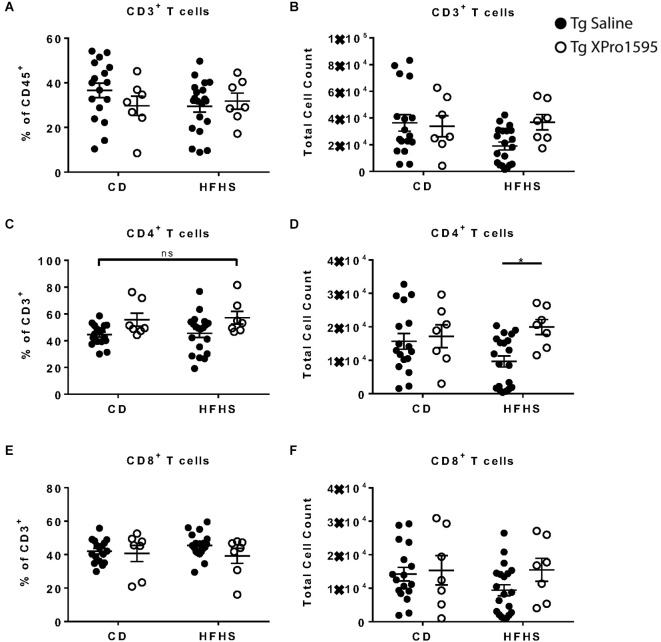
Inhibition of sTNF increases peripheral blood CD4^+^ T cell count in HFHS-fed 5xFAD Tg mice. **(A)** CD3^+^ T cell frequency of CD45^+^ immune cells (*n* = 7–20 mice per group). **(B)** CD3+ T cell number (*n* = 7–20 mice per group). **(C)** CD4^+^ T cell frequency of CD3^+^ T cells (*n* = 7–20 mice per group). **(D)** CD4^+^ T cell count (*n* = 7–20 mice per group). **(E)** CD8^+^ T cell frequency of CD3^+^ T cells (*n* = 7–20 mice per group). **(F)** CD8^+^ T cell count (*n* = 7–20 mice per group). Asterisks indicate significant (*p* < 0.05) differences by Tukey’s multiple comparisons test between groups following two-way ANOVA. Tg saline (black circle), Tg XPro1595 (white circle). ns = not significant.

In peripheral blood immune cells from the 5xFAD mice in this study (Supplementary Figure S1: Flow gating strategy), T cell numbers and frequencies ranged widely. No significant effect of diet was found on CD3^+^, CD4^+^, or CD8^+^ T cell populations (Figures 3A–F). Inhibition of sTNF with XPro1595 did influence CD4^+^ T cell populations (main effect of treatment on the frequency of T cells *p* = 0.004 and on the number of T cells *p* = 0.028) and significantly increased the CD4^+^ T cell number in Tg mice on the HFHS to an average closer to that of mice on CD (Figures 3C,D).

**Figure 4:**
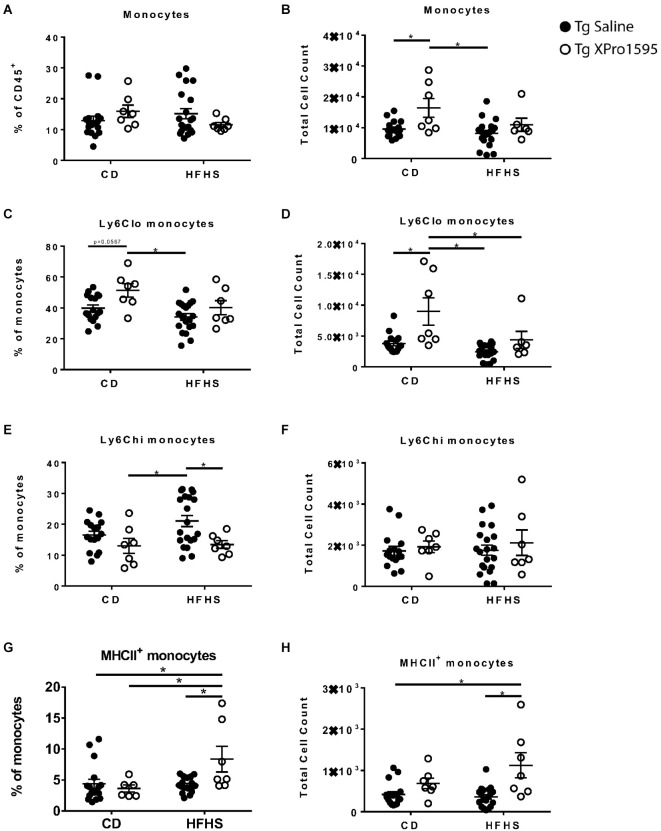
Effects of HFHS diet and sTNF inhibition on myeloid populations in the blood of 5xFAD female mice. **(A)** Monocyte frequency of CD45^+^ immune cells (*n* = 17–20 mice per group) in the blood of Tg mice on CD or HFHS diet. **(B)** Monocyte count (*n* = 17–20 mice per group). **(C)** Ly6C^lo^ frequency of monocytes (*n* = 17–20 mice per group). **(D)** Ly6C^lo^ monocytes count (*n* = 17–20 mice per group). **(E)** Ly6C^hi^ frequency of monocytes (*n* = 17–20 mice per group). **(F)** Ly6C^hi^ monocytes count (*n* = 17–20 mice per group). **(G)** MHCII^+^ monocyte frequency (*n* = 17–20 mice per group). **(H)** MHCII^+^ monocytes count (*n* = 17–20 mice per group). Asterisks indicate significant (*p* < 0.05) differences by Tukey’s multiple comparisons test between groups following two-way ANOVA. Tg saline (black circle), Tg XPro1595 (white circle).

The average number but not frequency of peripheral blood monocytes was lower in 5xFAD mice fed HFHS diet (main effect *p* = 0.028) while mice treated with XPro1595 had higher average numbers of monocytes compared with saline-treated mice (main effect *p* = 0.003; Figures 4A,B). This finding was primarily driven by changes in the population of monocytes expressing lower levels of Ly-6C (Figures 4C,D). Inhibition of soluble TNF also reduced the frequency of monocytes which expressed high levels of Ly-6C (main effect *p* = 0.0084), particularly in 5xFAD mice on an HFHS diet, which had a significantly lower percentage of Ly-6C^hi^ monocytes when treated with XPro1595 (Figures 4E,F). The combination of HFHS diet and soluble TNF inhibition resulted in higher levels of monocytes expressing MHC-II compared with the other groups in this study, while diet and treatment individually had minimal effect on this cell population (interaction of diet and treatment *p* < 0.05 for both frequency and counts; Figures 4G,H).

### 3.3 Diet and inhibition of sTNF with Xpro1595 induce changes in gut microbiota composition

**Figure 5:**
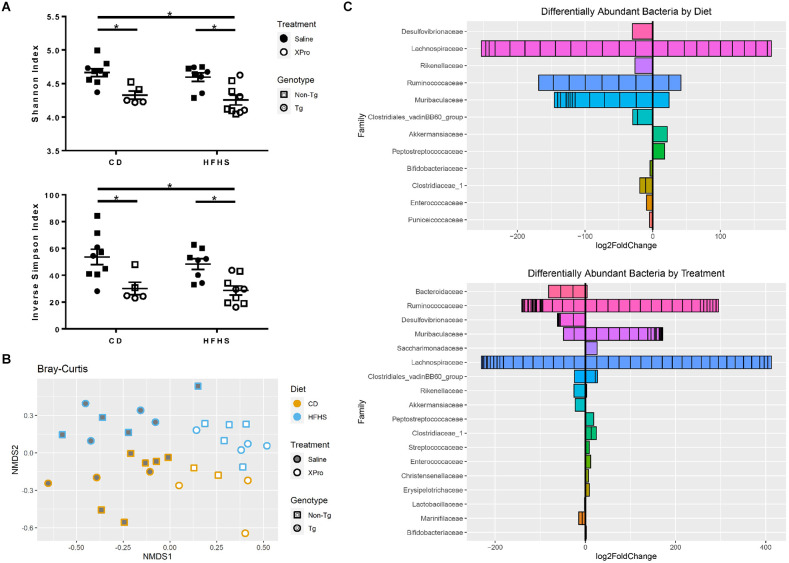
HFHS diet and XPro1595 significantly impacted gut bacterial populations of Tg and non-Tg mice. **(A)** Alpha diversity of stool bacterial communities (*n* = 5–9 mice per group). Asterisks indicate significant (*p* < 0.05) differences by Tukey’s multiple comparisons test between groups following two-way ANOVA (genotypes combined). **(B)** Non-metric multidimensional scaling (NMDS) using Bray-Curtis dissimilarity representing bacterial taxa composition by diet, treatment, and genotype (*n* = 31 mice total). **(C)** Log2 fold changes in the taxa grouped by a family that were identified as differing significantly in abundance with HFHS diet accounting for treatment and with XPro1595 treatment accounting for diet. Each subdivision of a horizontal bar represents a different taxon within the family, and the families are ordered according to adjusted *p*-value with the lowest at the top of the plot. Tg mice (circles), non-Tg mice (squares), saline (black-fill shape), XPro1595 (white-fill shape).

Given the selective effects of sTNF inhibition on alterations in peripheral immune cell profiles induced by a high-caloric diet, we investigated the extent to which this diet and sTNF inhibition disrupted the gut microbiome. Alpha diversity reflects the richness and evenness of taxa in the environment. While diet did not produce significant changes in alpha diversity in this study, there was a significant reduction in both Shannon (*p* < 0.0001) and inverse Simpson (*p* = 0.0001) indices with XPro1595 treatment as evaluated by two-way ANOVA (genotypes combined; Figure 5A). Linear regression confirmed that XPro1595 treatment reduced alpha diversity significantly (Shannon: *p* = 3.38 × 10^–5^, inverse Simpson: *p* = 1.15 × 10^–4^) when diet and genotype were taken into account and that diet and genotype were not associated with alpha diversity. There were also significant differences in the composition of bacterial communities (beta diversity) based on XPro1595 treatment (*p* < 0.001) and based on diet (*p* < 0.001; Figure 5B); genotype did not have a significant effect on beta diversity.

After accounting for treatment, the relative abundances of 57 taxa belonging to 16 classified genera and 12 classified families were found to differ significantly from HFHS diet (Figure 5C). These included increases in Akkermansiaceae (genus *Akkermansia*) and Peptostreptococcaceae and decreases in Desulfovibrionaceae, Rikenellaceae, Bifidobacteriaceae (genus *Bifidobacterium*), Clostridiaceae (genus *Clostridium* *sensu stricto*), Clostridiales vadin BB60, Enterococcaceae (genus *Enterococcus*), and Puniceicoccaceae. With the exception of one amplicon sequence variant (ASV) from the genus *Muribaculum* which was only found in a few HFHS diet-fed mice, other ASVs in the Muribaculaceae family decreased in abundance with an HFHS diet. There were increases and decreases in abundance of different ASVs in the Lachnospiraceae and Ruminococcaceae families with HFHS diet. Bacteria in the genus *Ruminococcaceae*_UCG-014 were less abundant with an HFHS diet, while several ASVs in the genus *Acetatifactor* (family Lachnospiraceae) were only detected in CD-fed mice.

After accounting for diet, the relative abundances of 124 taxa belonging to 32 classified genera and 18 classified families were found to differ significantly from XPro1595 treatment (Figure 5C). These included decreases in Akkermansiaceae (genus *Akkermansia*), Marinifilaceae, Lactobacillaceae (genus *Lactobacillus*), and Desulfovibrionaceae (including genera *Desulfovibrio* and *Bilophila*) with XPro1595 treatment. Bacteria in two ASVs in the genus *Odoribacter* were only detected in Saline-treated mice. There were increases in Clostridiaceae (genus *Clostridium* *sensu stricto*), Peptostreptococcaceae, Christensenellaceae, Streptococcaceae (genus *Lactococcus*), and Enterococcaceae (genus *Enterococcus*). The last two associations were driven by mice on the HFHS diet, as these ASVs were minimally represented among CD-fed mice. Contrastingly, an increase in Bifidobacteriaceae (genus *Bifidobacterium*) with XPro1595 treatment was driven by CD-fed mice. Bacteria from one ASV each in the families Erysipelotrichaceae (genus *Faecalibaculum*) and Saccharimonadaceae (genus *Candidatus* Saccharimonas) were detected in XPro1595-treated mice, but minimally in saline-treated mice. Many ASVs within the Muribaculaceae family increased in abundance with XPro1595; however, two, including an ASV from genus *Muribaculum*, were only found in a few saline-treated mice. Different ASVs within the families Bacteroidaceae, Ruminococcaceae, Lachnospiraceae, Clostridiales vadin BB60, and Rikenellaceae had significant increases and decreases in abundance by treatment. There was minimal detection of three related *Bacteroides* species, including *Bacteroides vulgatus*, in XPro1595-treated mice, while these species were common and abundant in saline-treated mice. A different *Bacteroides* ASV was more abundant in XPro1595-treated mice. Bacteria in the genera *Ruminococcaceae*_UCG-014 and *Alistipes* (family Rikenellaceae) were more abundant with XPro1595. Several related ASVs in the genus *Acetatifactor* (family Lachnospiraceae) were only detected in saline-treated mice.

### 3.4 HFHS diet and inhibition of sTNF alter CNS-associated T cell populations in 5xFAD mice

Changes in gut microbiome affect blood-brain barrier (BBB) permeability, adaptive immune cell development, and B and T cell phenotypes which can include trafficking to the CNS (Honda and Littman, 2016; Logsdon et al., 2018; Cowan, 2021). CNS-associated T cells are located primarily within the choroid plexus; however, T cells have been shown to infiltrate into the parenchyma of AD brains (Merlini et al., 2018). To analyze the effect of HFHS and sTNF inhibition on adaptive immune cell profiles in the CNS, we analyzed T cell populations in whole brain, including the choroid plexus, by flow cytometry.

**Figure 6:**
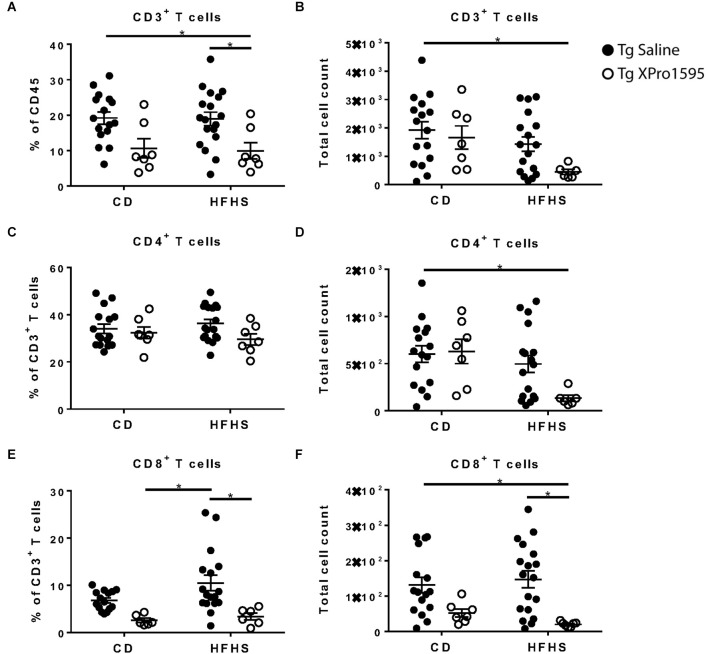
XPro1595 reduces CNS-associated CD8^+^ T cell populations in HFHS-fed 5xFAD mice. **(A)** CD3^+^ T cell frequency (*n* = 7–18 mice per group). **(B)** CD3^+^ T cell count (*n* = 7–18 mice per group). **(C)** CD4^+^ T cell frequency (*n* = 7–18 mice per group). **(D)** CD4^+^ T cell count (*n* = 7–18 mice per group). **(E)** CD8^+^ T cell frequency (*n* = 7–18 mice per group). **(F)** CD8^+^ T cell count (*n* = 7–18 mice per group). Asterisks indicate significant (*p* < 0.05) differences by Tukey’s multiple comparisons test between groups following two-way ANOVA. Tg saline (black circle), Tg XPro1595 (white circle).

Inhibition of sTNF was associated with a reduction in CD3^+^ T cell frequency (main effect *p* = 0.0005) while the HFHS diet was associated with a reduction in absolute T cell counts (main effect *p* = 0.0184). The combination of HFHS diet and XPro1595 treatment had the most pronounced effect, with T cell levels differing significantly from CD-fed saline-treated mice (Figures 6A,B. Within the T cell compartment, there was no significant effect of diet or treatment on CD4^+^ T cell frequency (Figure 6C), but the diet was associated with lower CD4^+^ T cell counts (main effect *p* = 0.0101), and the lowest counts were found in HFHS-fed XPro1595-treated mice (Figure 6D). A marked effect of the sTNF inhibition was observed in CD8^+^ T cells; among 5xFAD mice on the HFHS diet, those treated with XPro1595 had significantly lower levels of CD8^+^ T cells in the brain (Figures 6E,F).

### 3.5 HFHS diet in 5xFAD mice produces microgliosis in the hippocampus which is not entirely mitigated with sTNF inhibition

**Figure 7:**
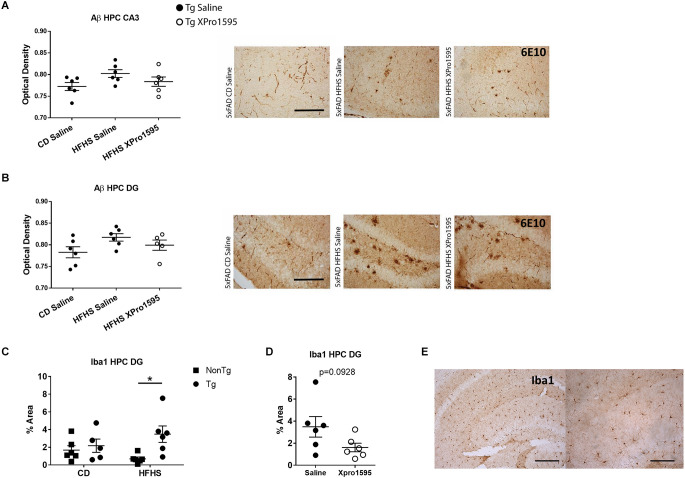
HFHS diet in 5xFAD mice increases Iba1 immunoreactivity in hippocampus with minimal effects of XPro1595 and minimal impact on Aβ. **(A)** Aβ (6E10) optical density in CA3 of 5xFAD hippocampus on CD and on HFHS diet +/– XPro1595 (*n* = 7 mice per group, one-way ANOVA) with representative images. **(B)** Aβ (6E10) optical density in dentate gyrus (DG) of 5xFAD hippocampus on CD and on HFHS diet +/– XPro1595 (*n* = 7 mice per group, one-way ANOVA) with representative images. **(C)** Iba1 staining coverage in the DG of the hippocampus in 5xFAD or non-Tg on CD vs. HFHS diet (*n* = 5–6 mice per group). **(D)** Iba1 staining coverage in the DG of the hippocampus in 5xFAD HFHS diet +/–XPro1595 (*n* = 6 mice per group). **(E)** Representative low (left) and high (right) magnification images of Iba1 staining in CA3 of the hippocampus of non-Tg on CD. non-Tg (square), Tg (circle), saline (black fill shape), XPro1595 (white fill shape). Scale bar in **(A,B)** = 500 microns; scale bar in **(E)** low magnification = 500 microns, high magnification = 200 microns. Asterisks indicate significant (*p* < 0.05) differences by Tukey’s multiple comparisons test between groups following two-way ANOVA. ns = not significant.

Next, we investigated the extent to which the HFHS diet exacerbated amyloid deposition and microgliosis and the ability of sTNF inhibition to block these processes. While immunohistochemical analysis suggested a trend for increased Aβ (6E10) load in hippocampal CA3 (Figure 7A) and DG (Figure 7B) in young 5xFAD mice on the HFHS diet which was somewhat reduced with sTNF inhibition, there was no significant difference between the groups. Similarly, no differences in Aβ load were found in hippocampal CA1 (data not shown). These results were confirmed using an alternative antibody against Aβ (Ab1–16) on separate tissue derived from a cohort of animals used in electrophysiological experiments (data not shown). In addition, this analysis revealed no obvious vascular deposition of Ab in any of the groups. Astrogliosis was unaltered in the hippocampus by the HFHS diet in non-Tg or in Tg mice (data not shown; CA1, DG, and CA3 GFAP % area). However, increased Iba1 staining was found in the DG of Tg mice on the HFHS diet as compared to non-Tg mice on the HFHS diet (Figure 7C; DG Iba1% area), but not CA3 or CA1 (data not shown). This increase in Iba1 was not significantly decreased by inhibition of sTNF; however, the data show a trend that is not statistically significant towards reduction in percent Iba1-immunoreactive area (Figures 7D,E; DG Iba1% area, unpaired *t*-test *p* = 0.0928). No effects of HFHS diet were found on mRNA expression associated with neuroinflammation in the hippocampus of Tg or non-Tg mice, including CCL2, TNF, CD45, and IL-6 (data not shown).

### 3.6 Inhibition of sTNF prevents HFHS-dependent deficits in LTP

**Figure 8:**
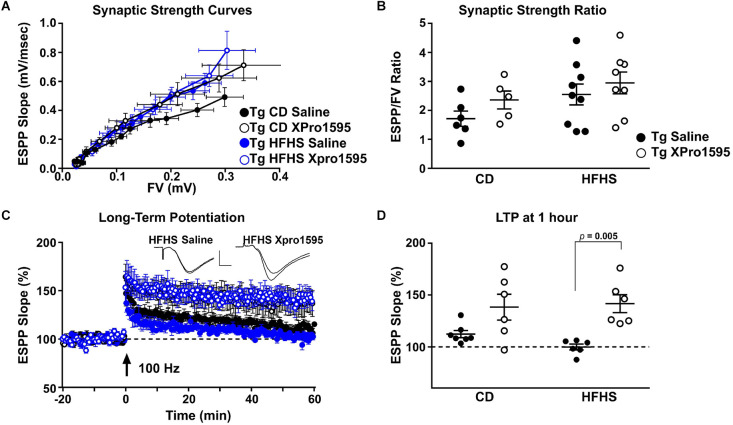
Soluble TNF inhibition with XPro1595 enhances LTP expression in 5xFAD mice. **(A)** Mean + SEM field EPSP (fEPSP) slope amplitudes plotted against mean + SEM presynaptic FV amplitudes across 12 stimulus intensities for Tg mice fed with CD or HFHS diet and treated with saline or XPro1595. **(B)** Scatterplot of maximal fEPSP/FV ratios in each diet/treatment group (*n* = 5–9 mice per group). **(C)** Time plot showing normalized fEPSP (mean + SEM) slopes recorded 20 min before and 60 min after the delivery of two 100 Hz stimulus trains (1 s duration, each train separated by 10 s). Insets show representative fEPSPs (before and 60 min after LTP induction) from HFHS diet mice treated with saline and HFHS diet mice treated with XPro1595. Calibration bars: 0.5 mV/5 msec. **(D)** Scatter plot showing LTP amplitudes for all animals at 60 min after the delivery of 100 Hz stimulation (*n* = 6–7 mice per group). Two-way ANOVA, Fisher’s LSD tests.

Although sTNF inhibition did not significantly affect the amyloid load, it was of interest to investigate the effects of sTNF inhibition on synaptic physiology. High-fat diets negatively affect synaptic function and plasticity in rodent models of amyloid pathology (Asadbegi et al., 2016; Lin et al., 2018; Salas et al., 2018). Previously, we observed LTP deficits in the hippocampus of Tg mice by 4–5 months of age and the ability of sTNF blockade to ameliorate age- and transgene-dependent synaptic dysfunction (MacPherson et al., 2017). Using similar electrophysiological approaches, we determined if synaptic deficits in hippocampal CA1 in 5xFAD mice were also modulated by the HFHS diet. Synaptic strength curves were constructed by plotting EPSP slope amplitudes relative to corresponding presynaptic FVs across increasing stimulus intensities. Treatment with XPro1595 with or without HFHS diet was associated with a leftward/upward shift in the synaptic strength curve relative to saline/CD treatment, suggesting an overall increase in synaptic strength (Figure 8A). However, while maximal EPSP/FV ratios were elevated by treatment with XPro1595 and/or HFHS diet, none of these differences reached statistical significance (Figure 8B). After synaptic strength curves were collected, stimulus intensity was adjusted to elicit a 1 mV EPSP, and field potentials were recorded over an additional 20 min baseline period before induction of LTP with high-frequency stimulation (Figure 8C). Though two-way ANOVA revealed no significant effect of diet on LTP amplitude, one sample *t*-tests showed that HFHS vehicle-treated mice were the only group that did not exhibit significant potentiation (i.e., > 100% of baseline; CD Saline, *p* < 0.05; CD XPro1595, *p* < 0.05; HFHS Saline, *p* = 0.94; HFHS XPro1595, *p* < 0.01). In contrast, XPro1595 treatment was associated with greater LTP levels regardless of diet treatment (two-way ANOVA; *F*_(1, 21)_ = 19.8, *p* < 0.001; Fisher’s LSD tests: CD Saline vs. CD XPro1595, *p* = 0.02; HFHS Saline vs. HFHS XPro1595, *p* = 0.001; Figure 8D). Other field potential parameters including population-spike threshold and paired pulse facilitation were unaffected by either diet or XPro1595 treatment (data not shown). These results demonstrate that the blockade of sTNF signaling with XPro1595 prevents AD-transgene or diet-induced synaptic plasticity deficits independent of changes in amyloid load.

## 4 Discussion

Human studies associate obesity and an overweight state with an increased risk of dementia and AD (Ma et al., 2020). In this study, the HFHS diet produced signs of metabolic syndrome in 5xFAD mice which were subtly impacted by the inhibition of sTNF with XPro1595. While XPro1595 treatment induced greater weight gain in young 5xFAD mice fed CD, mice fed HFHS diet gained comparable amounts of weight regardless of drug or vehicle treatment, and there was no effect of the drug on liver, cecum, or gonadal or retroperitoneal fat pad weight. Shortening of the intestine can occur under inflammatory conditions (Nordgren et al., 1997), and diets high in fat and sugar alter the gut environment and can induce a state reminiscent of subclinical inflammatory bowel disease (Arnone et al., 2021). In this study, we observed a significant reduction in small intestine length relative to body weight in saline-treated 5xFAD mice fed HFHS diet compared to CD, but XPro1595 treatment mitigated this effect, suggesting beneficial impacts of sTNF inhibition on the intestinal environment.

We previously reported that inhibition of sTNF impacts circulating cholesterol levels (De Sousa Rodrigues et al., 2019) and decreases liver triglycerides in wild-type mice. Consistent with our previous data, here we showed that sTNF inhibition decreased plasma cholesterol to levels indistinguishable from CD-fed mice. Evidence indicates a role of TNF on cholesterol metabolism (Kusnadi et al., 2019; Cardoso and Perucha, 2021), and increased circulating cholesterol is a known risk factor for AD (Giudetti et al., 2016; Wang et al., 2020). Future research will focus on the extent to which sTNF signaling can affect cholesterol metabolism and how this interaction can affect the risk for neurodegenerative disease.

There is a wealth of literature on the complex microbial modulation of extra-intestinal organs through immune-neuroendocrine interactions. It has been established that molecular signals from the gut contribute to microglia migration and homeostasis in the brain (reviewed in Butovsky and Weiner, 2018; Zheng et al., 2020). Microbiota-derived short-chain fatty acids (SCFA) have been implicated in this homeostatic signaling, and their levels may regulate the inflammatory milieu in the gut (Erny et al., 2015). Neurodegenerative diseases with chronic neuroinflammation have been reported to have features of microbial dysbiosis and microglial dysfunction (Abdel-Haq et al., 2019; Tansey et al., 2022). Indeed, in this study, we found that consumption of the HFHS diet by 5xFAD mice produced a marked increase in Iba1^+^ microglia (% area) in the dentate gyrus of the hippocampus. Additional studies will be needed to determine the extent to which dysfunction in gut microbiota-microglia interactions contributes to the pathogenesis and/or progression of these disorders.

Our novel findings that sTNF blockade may modulate gut-brain axis signaling *via* changes in gut microbiota and gut inflammation shed light on possible mechanisms driving gut-brain axis cross-talk in the context of neuro-immunity. Protective effects of sTNF blockade on the gastrointestinal system have been reported previously using XPro1595 in a model of gut inflammation (Chandrasekharan et al., 2013). This study examined the effects of XPro1595 treatment on the composition of the gut microbiota in the context of standard or HFHS diet. The powerful impact of a high-fat diet on bacterial populations has been extensively characterized (Bisanz et al., 2019; Guo et al., 2021), and we observed some changes with our HFHS diet that would be expected, such as decreases in *Muribaculaceae* and certain *Ruminococcaceae* taxa, which thrive on complex carbohydrate diets (Daniel et al., 2014; Lagkouvardos et al., 2019). Intriguingly, XPro1595 treatment also had marked effects on gut bacteria, reducing alpha diversity and producing marked changes in microbiota composition. Some of the changes in abundance of specific taxa that occurred with the HFHS diet appear to be shifted in the opposite direction with XPro1595; *Akkermansia* and *Muribaculum* were more abundant with the HFHS diet and less abundant with XPro1595, while *Bifidobacterium*, *Clostridium sensu stricto*, *Enterococcus*, *Ruminococcaceae*_UCG-014, and many *Muribaculaceae* species were reduced with HFHS diet and increased with XPro1595. Nonetheless, it is clear that XPro1595 treatment does not restore the overall HFHS diet microbiota to a composition resembling that of CD-fed mice. Rather, the inhibition of sTNF produces a distinct gut microbiome profile (Schierova et al., 2021). The functional implications of XPro1595-associated compositional shifts must be explored further in order to understand the full impact of sTNF inhibition in the context of an unhealthy diet and AD pathology. In this study, no differences in the effects of diet or XPro1595 treatment on gut bacterial populations by 5xFAD genotype were observed, but larger cohorts would be required to confirm this. While some studies have reported differences in the abundance of specific gut bacterial taxa in 5xFAD mice compared to WT (Brandscheid et al., 2017; Shukla et al., 2021), others have observed minimal difference in alpha and beta diversity measures (Parikh et al., 2020). In keeping with this, we found no differences in alpha or beta diversity by 5xFAD genotype in this study. Our findings do not preclude the possibility of an age- or sex-dependent genotype effect, however.

Interestingly, the present study showed central and peripheral immune cell alterations in 5xFAD mice with HFHS diet and with sTNF inhibition. XPro1595 increased levels of IL-4, IL-5, and IFNγ and decreased levels of CXCL1 in plasma. Effects on IL-5 and CXCL1 were less pronounced in mice fed HFHS diet, while sTNF inhibition increased plasma IL-2 concentrations, which were on average lower in HFHS-fed mice, to levels indistinguishable from CD-fed mice. sTNF inhibition had a similar effect on CD4^+^ T cells in HFHS-fed Tg mice, restoring counts which were somewhat lower in saline-treated HFHS-fed mice to CD levels. XPro1595 treatment also increased the number of MHCII^+^ monocytes in the blood. These findings suggest that sTNF inhibition in the context of the HFHS diet increases the potential for crosstalk between the adaptive (CD4^+^ T cells) and innate (MHCII^+^ monocytes) immune system. Furthermore, diet and sTNF inhibition had opposite effects on Ly-6C^lo^ monocyte populations; the HFHS diet decreased their levels while XPro1595 treatment increased them. These monocytes are traditionally considered to contribute to homeostatic functions and tissue repair (Geissmann et al., 2010). “Classical” Ly-6C^hi^ monocytes often play a role in responses to proinflammatory stimuli (Geissmann et al., 2010). sTNF inhibition significantly decreased their levels in HFHS-fed 5xFAD mice. HFHS diet reduced CD4^+^ T cell counts in the brain, and the lowest numbers were found in animals on the HFHS diet treated with XPro1595. sTNF inhibition was also clearly associated with a reduction in CD8^+^ T cell infiltration in the brain, with the greatest reduction observed in mice fed HFHS diet. While the role of cytotoxic T cells in AD is just beginning to be investigated, recent studies have discovered expanded CD8^+^ T cell populations in the brains of AD patients which may contribute to disease pathology (Gate et al., 2020; Unger et al., 2020). Our findings suggest that sTNF inhibition could suppress cytotoxic T-cell activity in the CNS. Further studies will be required to confirm the functional implications of these alterations in central and peripheral immune profiles.

Amyloid accumulation in the brains of these young 5xFAD mice induced by the HFHS diet was very modest, and XPro1595 had no significant effect on the modest increase. However, sTNF neutralization with XPro1595 in young Tg mice blocked the LTP deficits, suggesting that plaque burden alone is not what is driving synaptic dysfunction in young adult 5xFAD female mice. As a corollary, the XPro1595-mediated changes in the gut microbiome and CNS-associated CD8^+^ T cell number lead us to speculate that these two processes may in fact contribute to the observed improvement in synaptic function. Alternatively, the effects on synaptic function may be direct effects of sTNF signaling at the synapse (Beattie et al., 2002; Santello and Volterra, 2012; Maggio and Vlachos, 2018). Additional studies will be needed to distinguish between association and causality and the exact sTNF-dependent mechanisms responsible for the exacerbated effects of the gene-by-environment interplay.

In summary, our novel findings demonstrate that selective inhibition of sTNF signaling *via* XPro1595 likely has beneficial effects on immune profiles and mitigates long-term potentiation impairments in mice with a genetic predisposition to develop AD-like pathology fed an obesogenic diet. Future clinical trials in subjects at risk for AD due to chronic systemic inflammatory co-morbidities may allow us to investigate the extent to which these findings translate to the clinic.

## Data availability statement

The datasets presented in this study can be found in the National Center for Biotechnology Information (NCBI) BioProject repository, https://www.ncbi.nlm.nih.gov/bioproject/, accession PRJNA940119.

## Ethics statement

The animal study was reviewed and approved and all procedures were approved by the Institutional Animal Care and Use Committee of Emory University or University of Kentucky and complied with the National Institute of Health Guide for the Care and Use of Laboratory Animals.

## Author contributions

All authors listed have made a substantial direct and intellectual contribution to the work. KPM, LNE, BEW, JLG, MKH, MESR, LS, EMW, AMH, SDK, DLO, YY, and JC performed experiments. KPM, LNE, MCH, BEW, JLG, MKH, and MESR analyzed data. KPM, LNE, MCH, BEW, JLG, MKH, MESR, LS, EMW, and MGT drafted the manuscript and the figures. MGT and MCH edited the manuscript. All authors contributed to the article and approved the submitted version.

## Footnotes

^1^ http://www.rstudio.com/
