# Supplementary figures and images for: Soluble TNF mediates amyloid-independent, diet-induced alterations to immune and neuronal functions in an Alzheimer’s disease mouse model
Source: Front Cell Neurosci. 2023 Mar 15;17:895017. doi: 10.3389/fncel.2023.895017 (PMC10052573; doi:10.3389/fncel.2023.895017)

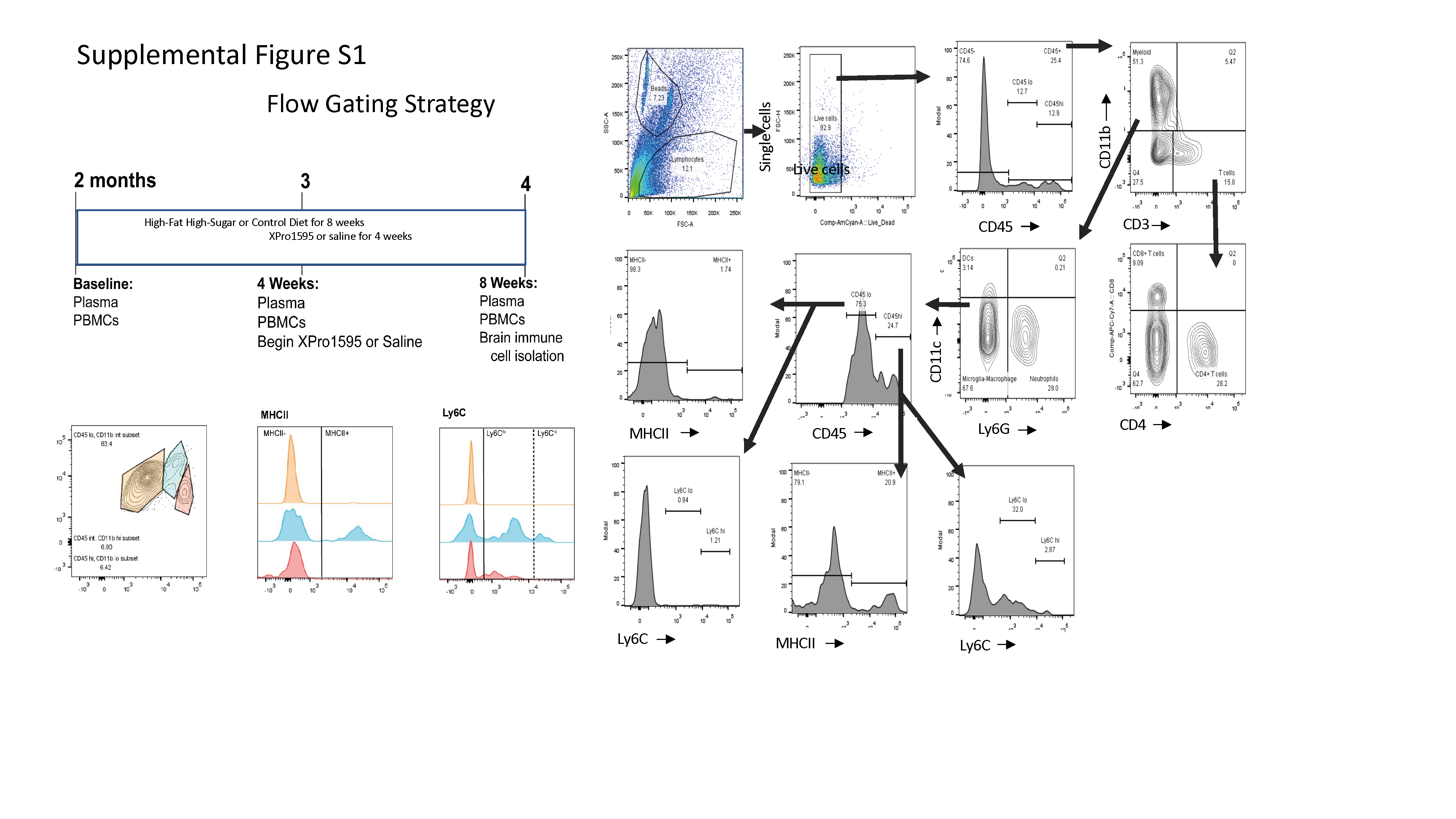

Supplement: Supplementary file 1 [file Image_1.TIFF]
